# Plasma cell disorders supress mucosal anti-bacterial immunity: another dimension of immunoparesis in plasma cell neoplasms

**DOI:** 10.1038/s41375-024-02398-1

**Published:** 2024-09-07

**Authors:** Sian Faustini, Y. L. Tracey Chan, Lilli Evans, Emily Collman, Alec Rapson, Claire Backhouse, Annabelle Emery, John P. Campbell, Sally Moore, Alex Richter, Guy Pratt, Mark T. Drayson, Jennifer L. J. Heaney

**Affiliations:** 1 Clinical Immunology Service, Institute of Immunology and Immunotherapy, University of Birmingham, Birmingham, UK; 2 University Hospitals Birmingham NHS Trust, Birmingham, UK; 3 Department for Health, University of Bath, Bath, UK; 4 Royal United Hospitals Bath NHS Foundation Trust, Bath, UK

**Keywords:** Myeloma, Immunology

## To the Editor:

Multiple myeloma (MM) is a cancer of plasma cells in the bone marrow associated with profound immunosuppression and high infection-related morbidity and mortality. In the first three months following diagnosis, serious bacterial infections affect a third of patients and infection contributes to half of all early mortality [1, 2]. *Streptococcus pneumoniae* is among the most common causes of infection and there is a two-hundred-fold increased risk of invasive pneumococcal disease in myeloma patients [3].

Diminished levels of polyclonal antibodies (immunoparesis) occur in 85% of MM patients at diagnosis [4] and both the presence and severity of immunoparesis are prognostic for survival [4, 5]. In addition to total levels of polyclonal immunoglobulin, pathogen-specific antibody levels could potentially impact patient outcomes. Antibodies against bacterial antigens relevant to common infections in MM have been shown to be markedly suppressed at diagnosis, which intensifies through therapy and renders patients in first remission without protective antibody levels across a range of pathogens [6].

COVID-19 has cast increasing attention beyond systemic antibodies to the role of mucosal immunity in preventing infections [7]. Salivary antibodies act in the first line of defence against many pathogens and may also help control carriage against specific bacteria, such as *Streptococcus pneumoniae* [8]. Consequently, the repertoire of antibodies in the oral environment may contribute to immune vulnerability and infection risk. This is particularly pertinent in patients with haematological malignancy who are already suffering from immunosuppression. Despite this, pathogen specific salivary antibody levels have yet to be characterised in such patients. Here we present a novel insight into oral immunity in patients with plasma cell dyscrasias, focusing on myeloma and related conditions, through assessment of pneumococcal specific anti-bacterial antibodies. Immunoparesis was analysed and confirmed conventionally in serum and then evaluated in saliva.

We evaluated serum and salivary antibodies in patients with plasma cell disorders (PCD cohort): active MM (patients either at diagnosis, undergoing therapy or relapse) and MM in remission and precursor conditions, monoclonal gammopathy of unknown significance (MGUS) and smouldering multiple myeloma (SMM). Patients (*N* = 62), median age 63 (32–90) years, were recruited from MM/MGUS clinics (University Hospitals Birmingham, UK and Royal United Hospitals Bath, UK). Of the patients with myeloma currently receiving treatment, therapies were lenalidomide and dexamethasone, cyclophosphamide and dexamethasone, VTD (bortezomib, thalidomide, and dexamethasone and lenalidomide maintenance). Healthy donors (HC cohort) representative of the age population from which PCDs typically arise were recruited from the local community, *N* = 40, median age 72 (63–83) years.

The study had ethical approval and all participants provided informed consent in line with the Declaration of Helsinki. Participants provided paired serum and saliva samples achieved through a four-minute passive drool. Samples were analysed using a multi-plexed bead assay that simultaneously measured antibody titres against 12 pneumococcal (Pn) serotypes (1, 3, 4, 5, 6B, 7 F, 9 V, 14, 18 C, 19 A, 19 F, 23 F) for both IgG and IgA, as previously described [9]. The World Health Organisation (WHO) defines a serum IgG protective antibody level as ≥0.35 μg/ml for a particular serotype, with this level on 8/12 serotypes recommended as the protective threshold based on vaccine studies [10]. Serum total immunoglobulins (IgG, IgA, IgM, The Binding Site, UK) were measured using a Cobas® 6000 Modular (Roche Hitachi) and classified as below the normal range based upon <5th percentile of adults aged ≥45 years in the UK (reported by Protein Reference Units): IgG <6 g/L; IgA <0.8 g/L; and IgM <0.5 g/L. Salivary flow rates affect immunoglobulin concentrations, therefore antibody secretion rates (saliva flow rate multiplied by concentration) are presented in main results/Figures to reflect the total availability of protein at the oral mucosal surface and control for hydration status.

## Confirmation of conventional and anti-bacterial antibody serum immunoparesis in the patient cohort

Immunoparesis of any total polyclonal immunoglobulin was observed in 50%, 87%, 85% and 75% among MGUS, SMM, active MM and MM in remission, respectively (Supplementary Table 1). Most MGUS and SMM patients had immunoparesis of a single immunoglobulin type, whereas most patients with active MM or MM in remission had immunoparesis of two immunoglobulin types. The rates of any immunoparesis in MGUS and SMM are slightly higher in this cohort study than statistics drawn from large registry or trial data bases [11, 12], likely reflecting differences in study size.

Of the different serotype combinations analysed, the WHO serum IgG pneumococcal threshold of ≥0.35 μg/ml was achieved in only 28% (210/744) of serotypes among the PCD cohort compared to 53% (253/480) of serotypes within the HC cohort (Table 1). Those achieving the protective threshold was significantly higher in the HC compared with the PCD cohort for 7/12 Pn serotypes.

**Table 1:** Number and proportion of plasma cell disorder patients and healthy donors with protective serum IgG antibody levels against bacterial antigens.

n (%)	PCD *n* = 62	MGUS *n* = 10	SMM *n* = 15	Active MM *n* = 21	MM remission *n* = 16	HC *n* = 40
Pn1	9 (15)***	2 (20)	3 (20)	3 (14)	1 (6)	19 (48)
Pn3	3 (5)**	1 (10)	0	1 (5)	1 (6)	10 (25)
Pn4	10 (16)	2 (20)	5 (33)	1 (5)	2 (13)	6 (15)
Pn5	18 (29)**	6 (60)	5 (33)	4 (19)	3 (19)	23 (58)
Pn6B	12 (19)	2 (20)	3 (20)	4 (19)	3 (19)	14 (35)
Pn7F	24 (39)	3 (30)	8 (53)	6 (29)	7 (44)	22 (55)
Pn9V	16 (26)	5 (50)	6 (40)	4 (19)	1 (6)	15 (38)
Pn14	35 (57)***	8 (80)	7 (47)	11 (52)	9 (56)	35 (88)
Pn18C	22 (36)***	5 (50)	7 (47)	7 (33)	3 (19)	30 (75)
Pn19A	23 (37)*	6 (60)	8 (53)	4 (19)	5 (31)	23 (58)
Pn19F	19 (31)	2 (20)	6 (40)	5 (24)	6 (38)	23 (58)
Pn23F	13 (21)*	4 (40)	3 (20)	3 (14)	3 (19)	17 (43)
8/12 serotypes with protective level	6 (10)***	1 (10)	3 (20)	1 (5)	1 (6)	16 (40)

Protection classed according to WHO cut-off of ≥0.35 μg/ml. Significance of PCD vs HC cohort indicated by ***p < .001, **p < .01, *p < .05, analysed using Chi Square. *PCD* plasma cell disorder cohort, *MGUS* monoclonal gammopathy of undetermined significance, *SMM* smouldering multiple myeloma, *MM* multiple myeloma, *HC* healthy control cohort, *Pn* pneumococcal.

The proportion of patients with protective levels for 8/12 Pn serotypes was notably low for all PCD patients (10%) compared to the HC cohort (40%), *p* < 0.001, with active MM and MM remission patients being the lowest (≤6%). This is consistent with a previous investigation that found MM clinical trial patients had supressed anti-pneumococcal antibodies at diagnosis, which then fall further post-induction therapy at 1 year from diagnosis [6].

## Serum anti-bacterial antibody immunoparesis in multiple myeloma extends to oral antibodies

**Fig. 1:**
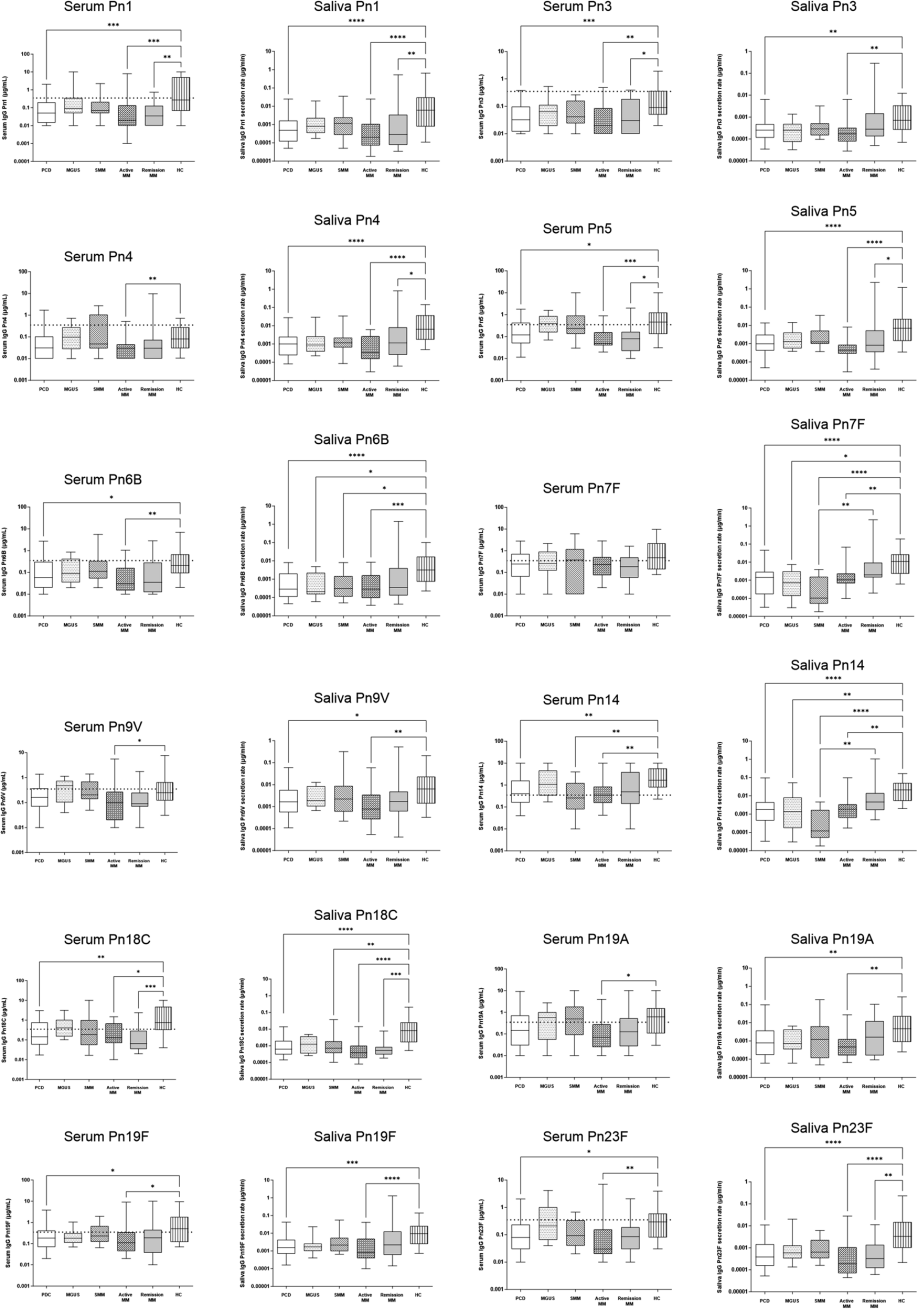
IgG anti-pneumococcal serum antibody concentrations and salivary antibody secretion rates in plasma cell disorder patients and healthy donors. Data is shown for 12 pneumococcal (Pn) serotypes. Data is presented for all plasma cell disorder (PCD) patients (*n* = 62) and then subdivided according to disease type/disease activity: MGUS (monoclonal gammopathy of unknown significance), SMM (smouldering multiple myeloma), and MM (multiple myeloma) patients with active disease or in remission. Data is also presented for the HC (healthy control) cohort (*n* = 40). Data was analysed using the Kruskal-Wallis test and Dunn’s multiple comparisons test. Significant differences between groups are indicated: *p < 0.05, **p < 0.01, ***p < 0.001. **** *p* < 0.0001. The dotted line on serum panels represents the protective cut-off (0.35 µg/mL). Lines indicate the median, boxes the 25–75th percentile and whiskers the 5-95th percentile.

IgG Pn antibody levels demonstrate the same patterns of immunoparesis within both serum and saliva (Fig. 1). For both systemic and oral compartments, descending Pn antibody levels were observed from MGUS to SMM to active MM, remaining supressed in post-therapy MM in remission. Significant differences between the PCD cohort and HC participants were seen for 8/12 serotypes and 12/12 serotypes in serum and saliva, respectively. These differences were driven by significantly lower antibodies in patients with active MM and MM in remission. Pn IgG levels in MGUS and SMM patients were only significantly different from that in HC for a minority of serotypes (serum Pn 14 and saliva Pn 6B, 7 F and 14). When assessing salivary antibodies based on IgG Pn protective levels in serum (≥0.35 μg/ml), patients without protection had lower corresponding Pn serotype salivary antibody secretion, which reached statistical significance for 6/12 serotypes (*p* < 0.01).

The parallel immunosuppression of serum and saliva Pn antibodies was also observed for IgA. Again, a descending pattern of Pn antibody levels occurred with the lowest in patients with active MM and MM in remission (Supplementary Fig. 1). Significantly lower Pn antibodies in patients with active myeloma and MM in remission compared to HC were seen for 12/12 and 9/12 serotypes, for serum and saliva, respectively. Except for salivary Pn14, MGUS or SMM did not significantly differ to HC. There were no significant differences within the PCD cohort, for either serum or saliva for IgG or IgA, between those <65 or ≥65 years old. Age was not examined in HC cohort as nearly all patients were ≥65 years.

To our knowledge, this is the first study to investigate pathogen specific serum and salivary antibodies in unison in the context of any haematological malignancy associated with immunodeficiency. Serum immunoparesis of polyclonal immunoglobulins have previously been investigated in relation to total saliva immunoglobulin levels. An earlier study of a similar number of MM and MGUS patients found evidence of IgG immunoparesis in serum being associated with lower salivary IgG secretion rates, but no relationship was observed between serum and saliva for IgA [13]. Differences may reflect the origin of antibodies, whereby the majority of total salivary immunoglobulins, particularly IgA, are known to be locally derived, whereas the reduction in Pn specific antibodies may represent a reduction in antibody primed from systemic responses.

It has been shown previously that Pn systemic responses are linked to local antibodies through both cross-sectional and vaccine studies. Vaccination with *Streptococcus pneumoniae* conjugate vaccines induces the development of mucosal antibodies and subsequent reduced Pn colonisation [8, 14]. Positive correlations between serum and saliva antibody concentrations for Pn serotypes has been demonstrated previously [8, 15], which was also observed in the present study (Supplementary Table 2).

The current study shows for the first time that MM patients with active disease or in remission after therapy have low levels of salivary IgG and IgA antibody levels against pneumococcus. Unlike serum Pn IgG, there are currently no clinical correlates of protection for salivary antibody levels to infer increased risk of infection but nevertheless reveal a novel dimension of immunoparesis for further exploration. Future larger healthy cohort studies could seek to identify cut-offs in saliva equivalent to the protective threshold in serum to help stratify the severity of oral immunoparesis, infection risk and relevance for patient outcomes.

In the UK, adults aged ≥65 years are eligible for PPV vaccination; however, vaccination history was not available for patients or HCs. It would be valuable to understand the impact of vaccination of salivary antibody levels and, for patients in remission following treatment, identify the optimal pneumococcal vaccine and its timing from both a systemic and mucosal antibody response to help protect patients against bacterial infections. Corroborating these findings in larger studies, and including both bacterial and viral antibodies, will help to further characterise mucosal immunity and infection risk in MM, and identify any relevant implications for clinical practice.

## Supplementary information

Supplementary

## Data Availability

Data in this Letter include patient data and are not available in a public repository. Data from the laboratory analysis included in this study are available on request.
