# Supplementary material for: Plasma cell disorders supress mucosal anti-bacterial immunity: another dimension of immunoparesis in plasma cell neoplasms
Source: Leukemia. 2024 Sep 7;38(11):2501–4. doi: 10.1038/s41375-024-02398-1 (PMC11519000; doi:10.1038/s41375-024-02398-1)
Supplement: Supplementary file 1 — Supplementary [file 41375_2024_2398_MOESM1_ESM.docx]

**Supplementary Figure 1. IgA anti-pneumococcal serum antibody concentrations and salivary antibody secretion rates in plasma cell disorder patients and healthy donors.** Data is shown for 12 pneumococcal (Pn) serotypes. Data is presented for all plasma cell disorder (PCD) patients and then subdivided according to disease type/disease activity: MGUS (monoclonal gammopathy of undetermined significance), SMM (smouldering multiple myeloma), and MM (multiple myeloma) patients with active disease or in remission. Data is also presented for the HC (healthy control) cohort. Data was analysed using the Kruskal-Wallis test and Dunn's multiple comparisons test. Significant differences between groups are indicated: **p* <.05, ***p* < .01, ****p*< .001. Lines indicate the median, boxes the 25-75th percentile and whiskers the 5-95th percentile.

**Supplementary Table 1.** Rates of serum immunoparesis among the plasma cell disorder patient cohort

| n (%) | **MGUS**  **n =10** | **SMM**  **n = 15** | **Active MM**  **n = 21** | **MM Remission**  **n = 16** |
| --- | --- | --- | --- | --- |
| Any immunoparesis | 5 (50) | 13 (87) | 18 (85) | 12 (75) |
| Immunoparesis of one polyclonal Ig | 4 (40) | 6 (40) | 7 (33) | 3 (19) |
| Immunoparesis of two polyclonal Ig | 1 (10) | 7 (47) | 8 (38) | 8 (50) |
| Immunoparesis of three polyclonal Ig  (light chain only and IgD m-protein) |  |  | 3 (14) | 1 (6) |

Ig= immunoglobulin, MGUS = monoclonal gammopathy of undetermined significance, SMM = smouldering multiple myeloma, and MM = multiple myeloma.

**Supplementary Table 2.** Spearman’s rank correlation coefficients (r^s^) for serum antibacterial antibody concentrations and saliva anti-bacterial antibody concentrations in patient and healthy control cohorts

| **r^s^** | **Concentrations of IgG in serum and IgG in saliva** | | **Concentration of IgA in serum and IgA in saliva** | |
| --- | --- | --- | --- | --- |
|  | **PCD** | **HC** | **PCD** | **HC** |
| Pn1 | .684*** | .830*** | .557*** | .680*** |
| Pn3 | .271* | .463** | .365** | .345* |
| Pn4 | .388** | .257 | .528*** | .363* |
| Pn5 | .489*** | .719*** | .364** | .365* |
| Pn6B | .475*** | .656*** | .607*** | 0.173 |
| Pn7F | .255* | .353* | .338** | .338** |
| Pn9V | .449*** | .254 | .481*** | .481*** |
| Pn14 | .439** | .330* | .457** | .457*** |
| Pn18C | .487*** | .678*** | .362** | .362** |
| Pn19A | .408** | .651*** | .615*** | .615*** |
| Pn19F | .419** | .541*** | .477** | .477*** |
| Pn23F | .558*** | .753*** | .384** | .384** |

PCD = plasma cell disorder cohort; HC = healthy control cohort. Pn = pneumococcal. Correlations were assessed separately; run for the PCD cohort and then again within the HC cohort. Significant positive correlations between serum and saliva concentrations are indicated: *** *p* < .001, ** *p* < .01, * *p* < 0.05. The same pattern of correlations was observed for both the PCD and HC groups; however, the strength of the correlation varied between cohorts for some stereotypes
